# Role of Mitochondria in Retinal Pigment Epithelial Aging and Degeneration

**DOI:** 10.3389/fragi.2022.926627

**Published:** 2022-07-15

**Authors:** Yao Tong, Zunyi Zhang, Shusheng Wang

**Affiliations:** ^1^ Department of Cell and Molecular Biology, Tulane University, New Orleans, LA, United States; ^2^ Department of Ophthalmology, Tulane University, New Orleans, LA, United States; ^3^ Tulane Personalized Health Institute, Tulane University, New Orleans, LA, United States

**Keywords:** RPE, aging, degeneration, mitochondria, senescense, cell death, age-related macula degeneration

## Abstract

Retinal pigment epithelial (RPE) cells form a monolayer between the neuroretina and choroid. It has multiple important functions, including acting as outer blood-retina barrier, maintaining the function of neuroretina and photoreceptors, participating in the visual cycle and regulating retinal immune response. Due to high oxidative stress environment, RPE cells are vulnerable to dysfunction, cellular senescence, and cell death, which underlies RPE aging and age-related diseases, including age-related macular degeneration (AMD). Mitochondria are the powerhouse of cells and a major source of cellular reactive oxygen species (ROS) that contribute to mitochondrial DNA damage, cell death, senescence, and age-related diseases. Mitochondria also undergo dynamic changes including fission/fusion, biogenesis and mitophagy for quality control in response to stresses. The role of mitochondria, especially mitochondrial dynamics, in RPE aging and age-related diseases, is still unclear. In this review, we summarize the current understanding of mitochondrial function, biogenesis and especially dynamics such as morphological changes and mitophagy in RPE aging and age-related RPE diseases, as well as in the biological processes of RPE cellular senescence and cell death. We also discuss the current preclinical and clinical research efforts to prevent or treat RPE degeneration by restoring mitochondrial function and dynamics.

## 1 Introduction

### 1.1 Functions of Retinal Pigmented Epithelial Cells

The RPE comprises a monolayer of polarized epithelial cells located in between the neuroretina and choroid. It functions as the outer blood-retina barrier and as a conduit for oxygen, nutrients, and waste between the neuroretina and choriocapillaris. RPE cells are highly polarized with regard to proteins and organelles distribution or secretion (Burke, 2008). On the apical side, microvilli of the RPE cells envelop and interact with photoreceptor outer segments (POS) of rods and cones; while on the basal side, the RPE cells display highly convoluted microfolds with close interaction with Bruch’s membrane (BrM) and the underlying choroid capillaries. RPE cells have many important functions including: 1) Maintaining function of the neuroretina. RPE cells synthesize and store melanin which absorbs reflected light that may otherwise degrade the visual image (Hu et al., 2008). 2) Providing a stable environment for RPE and nearby cells by maintaining the volume, ion concentrations and chemical composition of the subretinal space through transporters such as sodium/potassium adenosine triphosphatase (Na+/K+-ATPase) on RPE membrane (Wimmers et al., 2007). 3) Maintaining the function of photoreceptors. The POS regenerates every 7–12 days, RPE cells function to clear the old POS through phagocytosis, which protects photoreceptors from chronic oxidative stress exposure (Bok, 1985; Mazzoni et al., 2014). 4) Participating in the visual cycle. Through the process of visual cycle, retinoids cycle between the rod outer segments and the RPE. Light isomerizes 11-cis retinal into all-trans retinal causing visual pigment activation. RPE cells then take the photoproducts and regenerate 11-cis retinal before its returning to photoreceptors (McBee et al., 2001). 5) Regulating retinal immune response. Cytokines secreted by RPE, such as interleukin (IL)-1α, IL-1β, IL-7, tumor necrosis factor (TNF)-α, interferon (IFN)-γ, transforming growth factor (TGF)-β, play an important role in the homeostasis and inflammatory responses of the retina by activating resident immune cells and attracting circulating inflammatory cells (Holtkamp et al., 2001). Overall, RPE cells are fundamentally important for the metabolism and homeostasis of neuroretina. For more information of RPE function, refer to a review by Sparrrow et al. (Sparrow et al., 2010).

### 1.2 Overview of Cellular Senescence and Cell Death

Cellular senescence and cell death play important roles in aging and age-related diseases. Here we briefly summarize the two processes. Cellular senescence is a stable cell cycle arrest, which involves metabolic reprogramming, chromatin rearrangement and autophagy modulation (Kuilman et al., 2010). Senescent cells present enlarged cell size, increased reactive oxygen species (ROS) levels, persistent DNA damage response, arrested growth, apoptosis resistance, disorganized chromatin and changed gene expression (Chen et al., 2000; Hampel et al., 2004; Herbig et al., 2004). They also release chemokines, cytokines, proteases, and growth factors, which is collectively called senescence-associated secretory phenotype that would affect neighboring cells (Nelson et al., 2012). The accumulation of senescent cells could drive aging and age-related diseases (Childs et al., 2015). Multiple types of cell death could exist in RPE cells. Apoptosis is a classic type of programmed cell death which is regulated by the caspase family of proteins. Regulated necrosis also happens in RPE cells which includes but not limited to necroptosis, pyroptosis and ferroptosis. Necroptosis is morphologically characterized by cell swelling and bursting, associated with the release of intracellular contents (Hanus et al., 2013; Hanus et al., 2016). Activation of necrosomes is a marker of necroptosis. Pyroptosis is mediated by inflammasome activation and release of proinflammatory intracellular contents, including IL-1β and IL-18. Ferroptosis is characterized by lipid peroxidation and iron involvement. Ferroptotic cells usually do not have typical morphological characteristics of necrosis, but display mitochondrial shrinkage, increased mitochondrial membrane density and reduced mitochondrial cristae (Yagoda et al., 2007; Yang and Stockwell, 2008). For more in-depth review of cell death and senescence in RPE cells, please refer to our recent reviews (Hanus et al., 2015; Tong and Wang, 2020).

### 1.3 Mitochondrial Functions and Dynamics

Mitochondria are double membrane organelles in the cell responsible for energy production. Electron transport chain (ETC) and adenosine triphosphate (ATP) synthase are located within mitochondrial inner membrane, while enzymes of the tricarboxylic acid (TCA) cycle and fatty acid oxidation are in the matrix. Thus, mitochondria are very important for cellular energy metabolism, generating the majority of cellular ATP in eukaryotes (Wallace, 2009). Mitochondrial ATP production and membrane potential require the universal cofactor nicotinamide adenine dinucleotide (NAD). As an essential coenzyme, NAD gains two electrons and a proton from substrates at multiple TCA cycle steps, being reduced to NADH. An optimal NAD/NADH ratio is essential for efficient mitochondrial metabolism as TCA cycle ETC require NAD and NADH respectively (Stein and Imai, 2012). Mitochondria can provide energy to transport calcium (Ca^2+^) against its concentration gradient. They also modulate Ca^2+^ concentrations in the cytosol, sequestering the ion in the mitochondrial matrix which helps to maintain the appropriate concentrations of Ca^2+^ inside the endoplasmic reticulum (ER) and near the sites of exocytosis (Rizzuto et al., 2012; Finkel et al., 2015). Mitochondria also provide activation signals, such as mitochondrial ROS and oxidized mitochondrial DNA (mtDNA), and structural platform for inflammasome assembly and activation (Shimada et al., 2012; Elliott et al., 2018). What’s more, mitochondria can regulate ketone body formation, heme biosynthesis and the urea cycle (Boyman et al., 2020). In addition, mitochondria are also responsible for producing cell signaling molecules (Boyman et al., 2020), and many cellular processes including cellular senescence and cell death (Davalli et al., 2016). Mitochondria are highly dynamic and undergo fusion/fission, biogenesis, and mitophagy in response to energy needs and stresses. Together, these constitute mitochondrial quality control, which is essential for maintaining mitochondrial homeostasis and function (Meyer et al., 2017; Ren et al., 2020). The processes that are involved in mitochondrial quality control include:

1) **Mitochondrial fusion** is the process by which the outer membrane guanosine triphosphatases (GTPase) proteins mitofusins (MFN) fuse two outer mitochondrial membranes and the inner membrane GTPase optic atrophy 1 (OPA1) fuses two mitochondrial inner membranes to form one mitochondrion. It is a potential mitochondrial repair mechanism through the diffusion of mtDNA and proteins (van der Bliek et al., 2013; Tilokani et al., 2018).
2) **Mitochondrial fission** increases the number of mitochondria. In this process, dynamin-related protein 1 (DRP1) is recruited by mitochondrial outer membrane proteins including mitochondrial fission factor, and forms a ring around the mitochondrion, clinching it to eventually form two separate mitochondria. Mitochondrial fission is a regular event during cell division and can be induced by oxidative stress and DNA damage (van der Bliek et al., 2013; Tilokani et al., 2018). Defective or imbalanced mitochondrial fission/fusion may affect mitochondrial motility and energy production, promote oxidative stress and mtDNA deletion, and impair Ca^2+^ buffering, all of which could lead to cell death (Chan, 2012).
3) **Mitochondrial biogenesis** is the process of mitochondrial self-replication, involving replication and expression of mtDNA-encoded genes, as well as the synthesis and import of nuclear-encoded mitochondrial genes (Ploumi et al., 2017). It occurs in response to the energy demand triggered by developmental signals and environmental stressors. Peroxisome proliferator-activated receptor gamma coactivator (PGC)-1α and nuclear respiratory factors (NRFs) are key regulators of mitochondrial biogenesis. The interplay between NRF2 and PGC-1α through their interaction and regulation loop collectively controls mitochondrial biogenesis (Gureev et al., 2019).
4) **Mitophagy** is a selective engulfment process of dysfunctional mitochondria in lysosome by autophagy under adverse conditions, such as oxidative stress, hypoxia, mitochondrial membrane potential loss, accumulation of unfolded protein and iron starvation (Palikaras et al., 2018). Mitophagy can occur through different pathways based on the targeting signals on damaged mitochondria that initiate mitophagy: 1) Ubiquitin-dependent mitophagy: a) Parkin dependent: PTEN-induced putative kinase 1 (Pink1)-Parkin pathway; b) Parkin independent but ubiquitin dependent mitophagy; 2) Ubiquitin-independent or receptor based mitophagy: a) Apoptosis related proteins as mitophagy receptors or inhibitors; b) Other mitophagy receptors; 3) Lipid based mitophagy: a) Cardiolipin based; b) Sphingolipid based; 4) Micromitophagy. Pink1-Parkin pathway is the best-studied mitophagy pathway among all others. Pink1 becomes lodged in the translocase of the outer membrane (TOM) and leads to the recruitment of Parkin, an E3 ubiquitin ligase, which polyubiquitinates proteins on the outer mitochondrial membrane and triggers the recruitment of autophagy receptors and autophagy machinery to degrade the mitochondrion (Narendra et al., 2008; Youle and Narendra, 2011). The adenosine5′-monophosphate-activated protein kinase (AMPK) is recently described as a master sensor of cell stress and is emerging as a crucial regulatory factor of mitochondrial metabolism and mitophagy. It has been reported that AMPK plays a role in mitochondrial fission and autophagosomal engulfment, and interplays with Pink1-Parkin signaling (Herzig and Shaw, 2018).

As a part of the quality control mechanism, mitophagy enables the degradation of damaged and superfluous mitochondria, which prevent detrimental effects and reinstates cellular homeostasis in response to stress. Mitochondrial fission facilitates mitophagy by dividing mitochondria into fragments or segregating damaged mitochondrial subdomains for autophagosome engulfment, therefore promoting mitophagy. Impaired mitophagy indicates less mitochondrial turnover, which leads to the accumulation of dysfunctional senescent mitochondria and age-related disorders (Doblado et al., 2021). The ubiquitin proteasome system (UPS) can ubiquitinate mitochondrial proteins via a cascade of E1, E2, and E3 enzymes, and redirect them for proteasome degradation (Livnat-Levanon and Glickman, 2011). The importance of UPS in mitophagy largely attributes to the E3 ligase Parkin. Ubiquitin-dependent degradation of key mitochondrial proteins also participates in regulating mitochondrial energy metabolism, including regulating the turnover of several oxidative phosphorylation (OXPHOS) proteins.

In summary, mitochondria are critical for energy production and the integrity and quality control of mitochondria are important for cellular processes in response to different stresses.

### 1.4 Current Mitochondrial Assays Used in Research

Many methods have been used to assess the parameters of mitochondrial morphology, function, mtDNA and mitochondrial protein damage, mitochondrial metabolism and autophagy regulation to evaluate mitochondrial quality: 1) Assessing autophagy and mitophagy: a) Autophagy: Given the importance of autophagy in maintaining healthy mitochondrial populations, microtubule-associated protein 1 light chain 3 (LC3)-II western blot and LC3 puncta imaging can be used to determine if aberrations in autophagy alter mitochondrial quality (Tanida et al., 2008). b) Mitochondrial morphology: Confocal microscopy can be used to measure both the changes of morphology of the mitochondrial network under stresses, and mitophagy. The mitochondrial network can change primarily through fission or fusion. The level of fission/fragmentation in response to stress can be measured by quantifying the length of a cell’s mitochondrial population (Mitra and Lippincott-Schwartz, 2010). Transmission electron microscopy (TEM) can be used to obtain high-resolution micrographs of mitochondria, but mitochondrial ultrastructure (Tobias et al., 2018). c) Mitophagy: Mitophagy events can be determined from the co-localization between MitoTracker (MT) red (a red-fluorescent dye that stains mitochondria in live cells) and LysoTracker (LT) green (a cell-permeable green dye that stains lysosome in live cells). Imaging MT/LT co-localization can be performed to assess the increase or decrease of flux (Dolman et al., 2013). MitoTimer is another fluorescent reporter protein that can detect mitochondrial turnover within cells. It encodes a mitochondria-targeted green fluorescent protein when newly synthesized, which shifts irreversibly to red fluorescence when oxidized (Laker et al., 2014; Gottlieb and Stotland, 2015). Mito-Keima is a pH-sensitive, dual-excitation ratiometric fluorescent protein that can detect the delivery of mitochondria to the lysosome. In the alkaline environment, the shorter-wavelength (green) excitation predominates, while within the acidic lysosome, the Keima protein gradually shifts to the longer-wavelength (red) excitation, with partial overlap in the emission spectra. These properties of mito-Keima can be used to determine whether Keima-tagged mitochondria are at the physiological pH of the mitochondria (pH 8.0) or the lysosome (pH 4.5) (Sun et al., 2017). d) Mitochondrial mass protein levels and mtDNA copy: The protein complexes in the mitochondria can be turned over at different rates and a decrease in mitochondrial quality can be detected as decreased specific activity of the mitochondrial enzymes and damaged mtDNA. The levels of all five mitochondrial complexes can be assessed by measuring representative subunits from each complex which can be done using separate antibodies and probing each complex individually. This approach can be extended to evaluate levels of mitochondrial proteins involved in other metabolic pathways such as the TCA cycle. Measurement of mtDNA copy number can be used as an additional indicator of mitochondrial mass which can be done by real-time PCR using mtDNA directed primers; 2) Assessing metabolism: Metabolomics mass spectrometry can be used to analyze TCA cycle; glycolysis and glutaminolysis (Gowda and Djukovic, 2014); 3) Assessing mitochondrial bioenergetic function: The development of seahorse extracellular flux technology has allowed for the high throughput measurement of cellular bioenergetics and the activity of individual mitochondrial complexes within the cell and in isolated mitochondria (Dranka et al., 2011; Salabei et al., 2014).

## 2 Retinal Pigmented Epithelial Structural and Functional Changes During Aging

**FIGURE 1:**
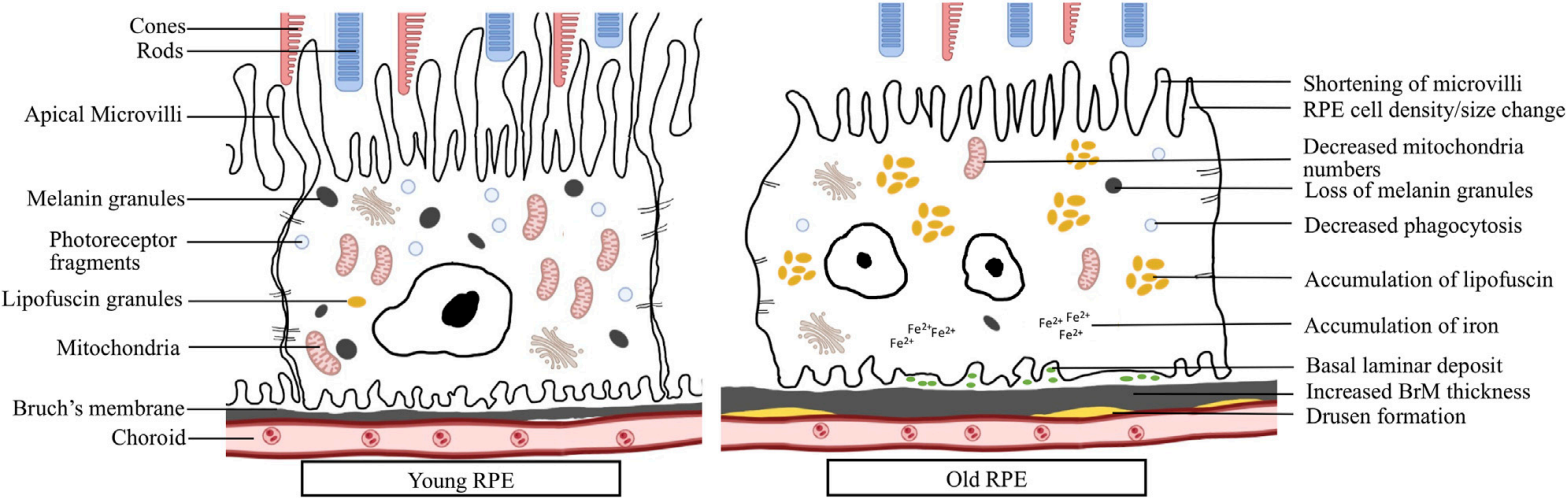
RPE changes during aging: Young RPE cell shows elongated microvilli, tight contact with nearby cells, containing plenty of mitochondria, melanin granules and photoreceptor fragments. Aged RPE cell shows larger size, multinucleation, shortened microvilli, decreased mitochondria numbers, loss of melanin granules, decreased phagocytosis, accumulation of lipofuscin and iron, basal laminar deposits, increased BrM thickness and accumulation of drusen.

The distinct functions of RPE cells make them susceptible to oxidative stress, due to their high metabolism and exposure to high oxygen, oxidized POS and polyunsaturated fatty acids (PUFAs). In addition, environmental factors, such as visible or ultraviolet (UV) light exposure and cigarette smoking, also pose oxidative stress to the RPE cells. These could lead to elevated levels of ROS and reactive nitrogen species in the cells, which can modify and damage carbohydrates, membrane lipids, proteins and nucleic acids, and eventually lead to pathological consequences. RPE cells are equipped with enzymatic and non-enzymatic antioxidative systems to protect against oxidative stress. However, the antioxidative capabilities diminish with aging, which leads to diminished RPE functions. RPE structural and functional changes associated with aging have been reviewed by Bonilha (Bonilha, 2008) and are summarized below (Table 1; Figure 1): 1) Aged RPE from human donors showed loss of melanin granules on their apical surface and accumulation of age pigment lipofuscin. Accumulation of secondary lysosomes and residual bodies containing lipofuscin, has been observed in post-mitotic and intermitotic cells during aging, and has been used as a universal index for cellular senescence (Schmucker and Sachs, 2002; Kubasik-Juraniec et al., 2004; Morales et al., 2004). 2) Shortening of RPE microvilli, which could affect some RPE key functions, such as phagocytosis of shed POS (Boulton and Dayhaw-Barker, 2001). 3) BrM is an acellular and pentalaminar structure formed by the RPE and choroid. Increased thickness, lipid content and advanced glycation products have been reported in the aging BrM, likely due to the entrapment of proteins and lipids in the extracellular matrix (Zarbin, 2004; Harris et al., 2017). Functionally, the elasticity of human BrM-choroid complex decreases linearly with aging. 4) Drusen are extracellular deposits of biomaterials below the RPE along BrM. Drusen can be originated from blood and/or RPE and is a clinical hallmark for age-related macular degeneration (AMD) and other age-related diseases including Alzheimer’s disease (Bergen et al., 2019). Drusen are ubiquitous in people over 50 years old and are considered as part of normal aging. 5) RPE cell density/size changes during aging. An earlier study using normal human donors showed that RPE density in the fovea decreased significantly by 0.3% per year with increasing age. An increase in RPE cell size and multinucleation has been observed in mice (Chen et al., 2016). 6) Age-related accumulation of iron in RPE/choroid has been observed in rats, while its increase in the retina was modest (Chen et al., 2009a; Chen et al., 2009b). Exposure of ARPE-19 cells (a spontaneously arising RPE cell line derived from the normal eyes of a 19-year-old male) to increased iron markedly decreased their phagocytosis activity, suggesting that iron accumulation with age may impair the phagocytosis and lysosomal functions of the RPE. In human, phagocytosis level in RPE from donors showed a moderate decrease with aging (Inana et al., 2018). 7) RPE secretome changes during aging. Secretome includes soluble proteins and proteins secreted as part of extracellular vesicles which function as mediators of cell-to-cell signaling and modulate cell activities. The secreted proteins could be used as biomarkers of some diseases. It has been reported that RPE secreted over 1,000 proteins, many of which change significantly due to ROS accumulated during aging (Meyer et al., 2019).

**TABLE 1:** RPE changes during normal aging and AMD.

RPE changes		References
Normal Aging	Loss of melanin granules	(Sarna et al., 2003; Kubasik-Juraniec et al., 2004)
	Lipofuscin accumulation	(Schmucker and Sachs, 2002; Kubasik-Juraniec et al., 2004)
	Decreased RPE cell density and increased RPE cell size and multinucleation	Chen et al. (2016)
	Shortening of RPE microvilli	Boulton and Dayhaw-Barker, (2001)
	Increased BrM thickness and decreased BrM/choroid elasticity	(Zarbin, 2004; Harris et al., 2017)
	Drusen formation	Bergen et al. (2019)
	Basal laminar deposit	van der Schaft et al. (1993)
	Accumulation of iron	Chen et al. (2009c)
	RPE secretome changes	Meyer et al. (2019)
	Modest decrease in RPE phagocytosis	Boulton and Dayhaw-Barker, (2001)
AMD	(May) have more cellular senescence	Tong and Wang, (2020)
	(May) have more cell death	
	Large soft drusen formation	Ambati et al. (2003)
	Hyper- or hypopigmentation in RPE	
	Lipofuscin aggregation in RPE	(Holz et al., 2001; Hwang et al., 2006; Ach et al., 2015)
	High variable and thicker RPE layer	Zanzottera et al. (2015)
	Shedding, dissociation and sloughing RPE cells (may indicate EMT process)	
	More significantly decreased RPE phagocytosis	Inana et al. (2018)
	Reduced mitochondrial function	(Saini et al., 2017; Ebeling et al., 2021)
	Increased inflammation markers	
	RPE secretome changes	(An et al., 2006; Meyer et al., 2019)

## 3 Retinal Pigmented Epithelial Changes in Age-Related Macular Degeneration

Aging, together with genetic and environmental factors, cause RPE dysfunction and degeneration, which significantly contributes to age-related retinal diseases including AMD. The RPE changes involve RPE functional decline, morphological changes, epithelial-mesenchymal transition (EMT), senescence and cell death. AMD is a leading cause of blindness among the elderly population. Late AMD has two forms, “dry” and “wet” AMD. Geographic atrophy (GA) is the advanced form of dry AMD. It is featured by the irreversible loss of the RPE, photoreceptors and choriocapillaris, which eventually lead to vision loss. The features and phenotypes of RPE in AMD have been under extensive studies by numerous groups. Here we summarize the RPE changes in AMD (Table 1; Figure 1): 1) Structural changes of RPE can be visualized by spectral domain optical coherence tomography (SD-OCT), autofluorescence imaging, combined with physiopathological analysis under electromicroscopy. Large soft drusen and hyper- or hypopigmentation in the RPE were identified as key features preceding RPE cell dysfunction and late form of AMD (Wang et al., 2003). Lipofuscin aggregation has been observed almost exclusively in AMD eyes, implying more cellular senescence in AMD (Ach et al., 2015). In an independent longitudinal study, increased fundus autofluorescence was observed preceding GA development, suggesting the involvement of cellular senescence and subsequent cell death in atrophic AMD (Holz et al., 2001; Hwang et al., 2006). 2) RPE layer becomes highly variable and overall thicker in both GA and wet AMD eyes, which could be explained by hypertrophy or cellular senescence (Zanzottera et al., 2015). 3) Shedding, dissociating and sloughing RPE cells were observed in GA, suggesting death, transdifferentiation or emigration of RPE cells (Zanzottera et al., 2015). Apoptosis of RPE cells in AMD was also detected by terminal deoxynucleotidyl transferase dUTP nick end labeling (TUNEL) assay, the most widely used method to detect fragmented DNA in apoptotic cells or tissue samples (Dunaief et al., 2002). However, cells may become TUNEL positive during other types of cell death. Therefore, more studies are needed to confirm the RPE cell death mechanisms in AMD, especially during the process of GA. 4) RPE phagocytosis, while showing a moderate decline with age, was significantly reduced in AMD RPE, more than expected during normal aging (Inana et al., 2018) 5) Induced pluripotent stem cell (iPSC)-RPE cells from homozygous ARMS2/HTRA1 risk genotype showed significantly higher complement and inflammatory factors expression, and iPSC-RPEs from complement factor H (CFH) (Y402H) risk genotype showed reduced mitochondrial function and increased inflammation markers (Saini et al., 2017; Ebeling et al., 2021). 6) By analyzing the secretome from RPE cells of AMD and age-matched control donors, RPE cells were found to secrete a variety of extracellular matrix proteins, complement factors, and protease inhibitors that have been reported to be major constituents of drusen (An et al., 2007). AMD RPE cells secrete more galectin 3 binding protein, fibronectin, clusterin, matrix metalloproteinase -2 and pigment epithelium derived factor, but less secreted protein acidic and rich in cysteine than RPE cells from age-matched healthy donors. Overall, significant progress has been made regarding the morphological changes and pathogenic mechanisms of RPE in AMD. More mechanistic studies of RPE in AMD, including molecular mechanism, the involvement of EMT, cell senescence and different types of cell death, are still needed.

## 4 Mitochondrial Changes During Retinal Pigmented Epithelial Aging

Numerous studies support the involvement of mitochondria in aging (Table 2). In the 1950s, Denham Harman proposed the free radical theory of aging, which he later expanded it to the mitochondrial free radical theory of aging. In this theory, free radicals produced by mitochondrial activities damage cellular components and lead to aging. Mitochondrial changes during aging include: 1) Morphological changes, such as abnormally rounded mitochondria (Shigenaga et al., 1994), decreased number of mitochondria (Yen et al., 1989), concurrent with decreases in mtDNA copy number and mitochondrial protein levels (Stocco et al., 1977). 2) Many studies have reported mitochondrial functional decline during aging, including lower oxidative capacity, reduced OXPHOS, decreased ATP production (Chistiakov et al., 2014). There is an average decline of 8% per decade in ATP producing capacity (Short et al., 2005). 3) Mitochondrial ROS significantly increases with aging and in aging-related diseases. Moderate ROS helps to maintain cellular energy production and to regulate mitochondrial protective signaling pathways, which increase lifespan in lower organisms (Giorgi et al., 2018). However, excess ROS are pathogenic and may induce cell degeneration or even cell death. For example, ROS and mitochondrial Ca^2+^ overload can cause the opening of the mitochondrial permeability transition pore, which leads to apoptosis (Rottenberg and Hoek, 2017). During normal aging, increased ROS level promotes oxidative damage to mitochondrial DNA, lipids, and proteins (Jarrett et al., 2008). 4) Impaired balance between mitochondrial fission and fusion is related to age-dependent decline in mitochondrial biogenesis. Mitochondrial fusion has beneficial effect to prolong lifespan by increasing bioenergetics efficiency, maintaining ATP production (Gomes et al., 2011; Rambold et al., 2011). Meanwhile, mitochondrial fission is associated with aging due to increased oxidative stress, mitochondrial depolarization, and reduced ATP production (Jheng et al., 2012). Fission maintains mitochondrial quality and integrity by involving in the selection of dysfunctional mitochondria. Mitophagy selectively removes defective mitochondria which have impaired oxidative capacity and declined integrity (Ding and Yin, 2012). With age, mitophagy was observed to decrease (Cavallini et al., 2007), which leads to an accumulation of damaged mitochondria, advanced oxidative stress, and increased apoptosis (Masiero and Sandri, 2010). 5) Mitochondrial density appears to decline gradually during aging, suggesting a decrease in mitochondrial biogenesis during aging, which could result from age-dependent reduction in levels of PGC-1*α* (Crane et al., 2010). 6) mtDNA damage, mutation and deletion during aging. mtDNA encodes essential components of OXPHOS and protein synthesis machinery (Falkenberg et al., 2007). Thus, oxidative stress-induced mtDNA damage and mutations impair either the assembly or the function of the respiratory chain which then trigger further accumulation of ROS which could be lethal (Hiona and Leeuwenburgh, 2008). Besides point mutations, deletions of mtDNA are detected at higher frequency in aged human and animal tissues (Nekhaeva et al., 2002; Kujoth et al., 2007). In addition, mtDNA abundance also declines with age in various tissues of human and rodent (Wallace, 2001; Miller et al., 2003; Welle et al., 2003). MtDNA containing unmethylated CpG dinucleotides and can trigger inflammation that aggravates tissue injury by activating toll-like receptor 9, inflammasomes, and the stimulator of interferon genes pathway (Nie et al., 2020). It has been reported to be pro-inflammatory in various diseases such as Alzheimer’s disease and heart failure (Wilkins et al., 2014; Nakayama and Otsu, 2018). Intracellular mtDNA has been shown to induce the secretion of inflammatory cytokines IL-6 and IL-8, and the priming of the NLR Family Pyrin Domain Containing 3 (NLRP3) inflammasome in RPE cells (Dib et al., 2015).

**TABLE 2:** Mitochondrial changes during normal aging and AMD.

Mitochondrial changes		References
Normal Aging (in general)	Abnormally round shape	Shigenaga et al. (1994)
	Reduction in mitochondrial number	Yen et al. (1989)
	mtDNA mutation, deletion and damage and reduced copy number	Falkenberg et al. (2007)
	Lower mitochondrial ATP level	Chistiakov et al. (2014)
	Reduced mitochondrial membrane potential	Rottenberg and Hoek, (2017)
	Decreased mitophagy	Cavallini et al. (2007)
	Increased mitochondrial ROS level	Giorgi et al. (2018)
	Impaired balance of mitochondrial fission and fusion balance (In most cases, more fission during aging and more fusion in longevity models)	(Gomes et al., 2011; Rambold et al., 2011) (Jheng et al., 2012)
	Decreased mitochondrial biogenesis	
RPE Aging	Round or oval in mitochondrial shape	(He et al., 2010) (Gouras et al., 2016)
	Disorganized mitochondrial cristae	
	Irregular in mitochondrial size	
	Sparse mitochondrial distribution in the cytoplasm	
	Reduction in mitochondrial number	
	mtDNA damage	Wang et al. (2008)
	Lower ATP production	(He et al., 2010) (Yako et al., 2021)
	Reduced mitochondrial membrane potential	Yako et al. (2021)
	Decreased cytoplasmic Ca^2+^ concentration	(Feher et al., 2006; Sridevi Gurubaran et al., 2020)
	Increased mitochondrial Ca^2+^ sequestration	(Karunadharma et al., 2010; Brown et al., 2019a)
	Impaired mitochondrial fission and fusion balance	Zhang et al. (2020)
AMD	Abnormal mitochondrial shape and size	(Hanus et al., 2016; Brown et al., 2019b; Tang et al., 2022)
	Reduction in mitochondrial number	(Feher et al., 2006; Sridevi Gurubaran et al., 2020)
	mtDNA damage	(Karunadharma et al., 2010; Brown et al., 2019a)
	Impaired mitochondrial fission and fusion balance	Yu et al. (2020)
	Lower mitochondrial ATP production	(Arselin et al., 2004; Bornhovd et al., 2006; Nordgaard et al., 2008; Terluk et al., 2015; Ferrington et al., 2017; Golestaneh et al., 2017)
	Reduced mitochondrial membrane potential	Yumnamcha et al. (2019)
	Lower basal respiration and maximum respiration	(Ferrington et al., 2017; Brown et al., 2019b; Ebeling et al., 2021)

**FIGURE 2:**
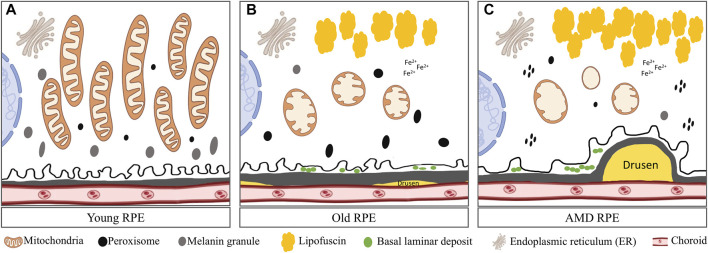
Comparison of mitochondria in young, aged and AMD RPE: **(A)** Young RPE cell contains numerous mitochondria with long axes, usually oriented from the apical to the basal surfaces of the RPE and are parallel to one another. The mitochondrial cristae are well preserved. Several peroxisomes appeared as small, round, electron-dense organelles. Plenty of melanin granules exist in the cells. **(B)** In aged RPE cell, mitochondria show membrane disorganization and loss of cristae. Accumulated lipofuscin presents in the cell. Several peroxisomes of various density, shape and size were distributed randomly in the cytoplasm. Less melanin granules appear in the cell. Also, small drusen forms underneath BrM and basal lamina deposits forms in between the cell and BrM. **(C)** In AMD RPE, advanced mitochondrial alterations occur. Most mitochondria had severe disorganization of membranes that varied from focal to complete loss of cristae. Peroxisomes are clustered and aggregated in the cell. Large and soft drusen forms underneath the BrM.

Only a few papers have reported the mitochondrial structural and functional changes during RPE aging in humans. Using electron microscopy, mitochondria in young RPE were found to be numerous, most bacillus-like shaped, rich in well preserved cristae, and orientated parallel with the apical-basal axis (Feher et al., 2006). In aged eyes, mitochondria of the RPE were decreased in number, variable in size, usually oval shaped, sometimes with disorganization of cristae (Figure 2). In another study, using isolated primary RPE cells from young (9–20)-, mid-age (48–60)-, and >60 (62–76)-year-old donors, some different morphological changes in mitochondria were observed. Mitochondria from the two younger groups were found to be numerous, regular in size, and with round or oval shapes (He et al., 2010). Cristae were distinctly visible and the outer membranes appeared to be intact. However, mitochondria from the >60 age group RPE cells were sparsely distributed in the cytoplasm, irregular in size, tubular in shape, larger, and with electron-dense matrices, less distinct cristae, and disrupted outer membranes. Length of the mitochondria in this group (Length/width ratio) was almost seven folds greater compared to the other ages. These mitochondrial abnormalities correlated with lower ATP levels, reduced mitochondrial membrane potential, decreased cytoplasmic Ca^2+^ concentration, and increased Ca^2+^sequestration in the mitochondria in cells with advanced aging (He et al., 2010).

There are several reports regarding age-related mitochondrial changes in animal models of RPE aging. Using monkeys up to 35 years old, Gouras et al. found decreased mitochondrial number and density, increased mitochondrial length and increased clustering of very elongated mitochondria during aging (Gouras et al., 2016). Using C57BL/6 mice (up to 22 months old) and rats (up to 26 months old), Wang et al. found an increased mtDNA damage in aged RPE and choroid (Wang et al., 2008). Yako et al. reported that in the RPE of 12-month-old C57BL/6J mice, mitochondrial number was decreased, but cristae width was increased compared to young mice (Yako et al., 2021). They also found that pharmacological suppression of mitochondrial fission improved POS phagocytosis, suggesting that mitochondrial dysfunction and fission in RPE could impede phagocytosis and cause retardation of the visual cycle. Metabolomics analysis in the RPE/choroid of young (6 weeks) and old (73 weeks) C57BL6/J mice identified 45 significantly changed metabolites, with most of which are involved in the mitochondrial metabolism, glucose metabolism and amino acid metabolism pathways (Wang Y et al., 2018). NAD and riboflavin (a precursor for flavin adenine dinucleotide) levels were reduced to less than half, but nicotinamide (substrate for NAD synthesis) and the substrates for mitochondrial metabolism such as pyruvate and dihydroxyacetone phosphate were accumulated to higher levels in the old mice, suggesting impaired mitochondrial energy metabolism during RPE/choroid aging. Taken together, the research on the mitochondrial changes in RPE aging is still limited, more systematic studies with even older groups, with focus on mitochondrial functions, mitochondrial dynamics and quality control, are still needed.

## 5 Retinal Pigmented Epithelial Mitochondrial Changes in Age-Related Macular Degeneration

Several studies have reported RPE mitochondrial changes in AMD (Table 2). A reduction in mitochondrial number has been observed in AMD donor RPE compared to age-matched controls (Feher et al., 2006). A proteomic study revealed decreased expression of ATP synthase subunits in AMD RPE (Nordgaard et al., 2008). ATP synthase complexes participate in OXPHOS and maintain mitochondrial morphology and mitochondrial membrane potential. Decreased expression of ATP synthase subunits with AMD could lead to defects in these critical mitochondrial functions (Arselin et al., 2004; Bornhovd et al., 2006). Another change in the mitochondrial proteome is the decreased expression of the mitochondrial heat shock protein (mtHsp70) in AMD RPE (Nordgaard et al., 2008). mtHsp70 functions as a molecular chaperone that regulates the ATP-dependent import of nuclear-encoded proteins into the mitochondrial matrix (Nordgaard et al., 2006; Nordgaard et al., 2008; Kaarniranta et al., 2009). Therefore, decreased mtHsp70 levels could be detrimental to overall mitochondrial function and limit energy production in AMD RPE. mtDNA damages increase with age, more mtDNA damage in the macula of human AMD RPE has been detected compared to age-matched controls (Karunadharma et al., 2010). The extent of RPE mtDNA damage correlates with AMD severity. Damage to the mtDNA could be mitochondrial genome-wide, including the region encoding the subunit of ETC and the D-loop, the site of initiation for mtDNA transcription and replication of one mtDNA strand (Karunadharma et al., 2010; Terluk et al., 2015). Damage to either the D-loop or region encoding the ETC proteins could lead to negative functional outcomes for the mitochondria (Terluk et al., 2015). The role of mitochondria in AMD was also studied using transmitochondrial cybrids (Miceli and Jazwinski, 2005; Nashine et al., 2017; Nashine et al., 2020). Transmitochondrial cybrids were created by fusing mitochondrial DNA-deficient APRE-19 cell line with platelets isolated from either AMD patients or age-matched normal subjects. Therefore, the cybrids had identical nuclei but different mitochondria. AMD cybrids showed: 1) reduced cell viability, lower mtDNA copy numbers, and downregulated mitochondrial replication/transcription genes and antioxidant enzyme genes; and 2) elevated levels of genes related to apoptosis, autophagy and ER stress along with increased mtDNA fragmentation and higher susceptibility to amyloid-β-induced toxicity compared to control cybrids. These studies support an important role for mitochondria in AMD.

Mitochondrial functions have also been studied using in the cultured RPE or iPSC-RPE cells derived from AMD donors. Ferrington et al. found that mitochondria of RPE from AMD donors have significantly declined functions such as lower basal respiration, ATP production, and maximum respiration compared to age-matched controls (Ferrington et al., 2017). There is no difference in mtDNA content when comparing healthy and AMD donors. In another study by Golestaneh et al., accumulation of lipid droplets and glycogen granules, damaged mitochondria, and increased autophagosomes were observed in cultured AMD RPE cells (Golestaneh et al., 2017). Decreased NAD+ and Sirtuin 1 (SIRT1), increased PGC-1α acetylation (inactive form), lower AMPK activity, and overactive mammalian target of rapamycin (mTOR) pathway were also observed in AMD RPE cells, suggesting RPE metabolic dysregulation in AMD (Zhang et al., 2020). Compared with normal RPE, AMD RPE exhibit increased susceptibility to oxidative stress, produce higher ROS levels under stress conditions, and showed reduced mitochondrial activity with decreased ATP production. Of note, Ferrington et al.‘s study showed that AMD RPE are more resistant to acute oxidative stress (Ferrington et al., 2017). iPSC-RPE cells have been derived from AMD patients, and reduced mitochondrial function has been observed in iPSC-RPE cells from the CFH Y402H risk genotype RPE (Ebeling et al., 2021).

Although no AMD mouse models can recapitulate human AMD phenotypes due to their lack of macula, AMD mouse models can provide some insight into the mechanism of AMD. Here we present several mouse models with connection to mitochondria. RPE-specific knockout of mitochondrial antioxidant enzyme MnSOD (or SOD2) resulted in reduced RPE function with age (Brown et al., 2019a). Less electron dense and swollen mitochondria, disorganized mitochondrial cristae, reduced ATP production, and a compensatory increase in glycolytic metabolism have been observed in the *SOD2* ^*−/−*^ mice, supporting that mitochondrial oxidative stress can lead to mitochondrial malfunction, RPE metabolic reprogramming and RPE dysfunction. In another model, global knockout of NRF2 and PGC-1α, the master regulators of antioxidant production and mitochondrial biogenesis, led to disturbed autophagy, an accumulation of drusen-like deposits, and the infiltration of Iba-1 positive immune cells mimicking clinical features of the dry AMD phenotype (Felszeghy et al., 2019). In the *NRF2/PGC-1α* ^*−/−*^ mice, a decline in viable mitochondria and higher mitochondrial damage were observed in the RPE cells. Damaged mitochondria were marked by Pink1/Parkin and autophagosomes with mitochondrial cargo. These data support that defective mitochondrial antioxidative system and biogenesis in the RPE could lead to AMD-like phenotypes (Wang K et al., 2018; Wang et al., 2019). For more information about RPE mitochondria in AMD, refer to reviews by Kaarniranta (Kaarniranta et al., 2020) and Blasiak (Blasiak et al., 2020).

## 6 Role of Mitochondria in Retinal Pigmented Epithelial Senescence

**FIGURE 3:**
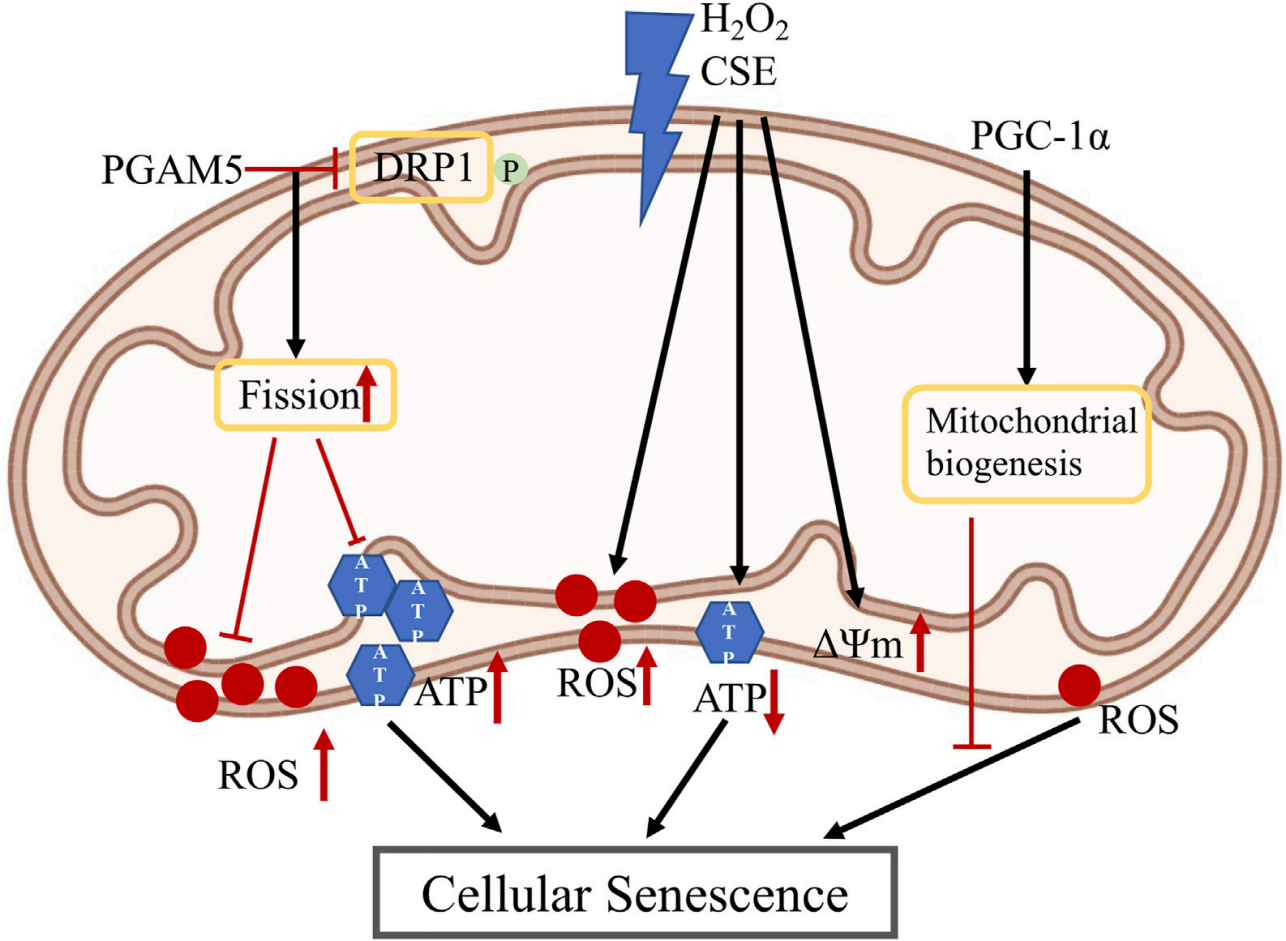
Mitochondria changes in RPE senescence: PGAM5 dephosphorylates DRP-1 which promotes mitochondrial fission, which then inhibits the increase of ROS and ATP in RPE cellular senescence; H_2_O_2_ and CSE induce increased mitochondrial ROS and membrane potential, also induce decreased ATP level which cause RPE cellular senescence; PGC-1α is a master regulator of mitochondria biogenesis and could reduce ROS level which may inhibit RPE cellular senescence. PGAM5: phosphoglycerate mutase 5; DRP1: dynamin-related protein 1; H_2_O_2_: hydrogen peroxide; CSE: cigarette smoke extract; PGC-1α: peroxisome proliferator-activated receptor gamma coactivator-1α; ATP: adenosine triphosphate; ROS: reactive oxygen species; ΔΨm: mitochondrial membrane potential.

**FIGURE 4:**
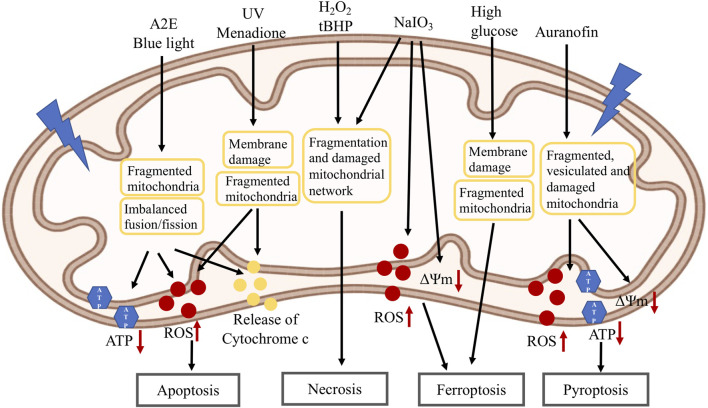
Mitochondria changes in RPE cell death induced by different stressors: A2E and blue light lead to fragmented mitochondria, imbalanced mitochondrial fusion/fission, decreased ATP level, increased ROS level and release of cytochrome C which then induce apoptosis; UV and menadione cause mitochondrial membrane damage, fragmented mitochondria, increased ROS level and release of cytochrome C which then induce apoptosis; H_2_O_2_, tBHP and NaIO_3_ cause fragmented mitochondria and damaged mitochondrial network, and lead to necrosis. NaIO_3_ also induces decreased mitochondrial membrane potential and increased ROS level, and leads to ferroptosis. High glucose induces mitochondrial membrane damage, fragmented mitochondria and cause ferroptosis; Auranofin causes decreased mitochondrial membrane potential, fragmented, vesiculated and damaged mitochondria, increased ROS level and decreased ATP level which lead to pyroptosis. A2E: N-retinylidene-N-retinyl-ethanolamine; UV: ultraviolet; H_2_O_2_: hydrogen peroxide; tBHP: tert-butyl hydroperoxide; NaIO_3_: sodium iodate; ATP: adenosine triphosphate; ROS: reactive oxygen species; ΔΨm: mitochondrial membrane potential.

Cellular senescence and cell death are important processes involved in aging and age-related diseases. Mitochondria have been established as important regulators of senescence and cell death. In this and next section, we summarize the role of mitochondria in RPE cellular senescence (Figure 3) and cell death (Figure 4).

Deregulation of mitochondrial homeostasis, shown by impaired mitochondrial biogenesis, metabolism and dynamics, has emerged as a hallmark of cellular senescence, which also drives the senescent phenotypes (Vasileiou et al., 2019). Defects in mitochondrial OXPHOS, reduced ATP production, and increased mitochondrial ROS production have been reported in cells undergoing senescence. In senescent cells, mitochondrial ROS can further aggravate cellular senescence by increasing telomere shortening, DNA damage and sustaining DNA damage response signaling pathway (Passos et al., 2010). In addition, mtDNA damage induced by ROS can impair OXPHOS function and further increase ROS release, forming a vicious cycle in aggravating senescence. Mitochondrial dynamics (including biogenesis, fusion, fission and mitophagy) are also linked to senescence. In aging endothelial cells, mitochondrial membrane potential, mitochondrial fusion and fission are reduced (Jendrach et al., 2005). In stress-induced senescent cells, highly elongated mitochondria with enhanced cristae structure have been reported (Yoon et al., 2006). Depletion of mitochondrial fission 1 protein (Fis1) caused sustained elongation of mitochondria and senescent phenotypes, whereas reintroduction of Fis1 protein restored mitochondrial fission and partially reversed the senescent phenotypes (Lee et al., 2007). Depletion of both Fis1 and mitochondrial fusion protein OPA1 resulted in mitochondrial fragmentation phenotypes and rescued senescence-associated changes. Intriguingly, sustained mitochondrial elongation is associated with reduced mitochondrial membrane potential, increased ROS level and DNA damage. These indicate that sustained mitochondrial elongation can trigger senescence-associated changes. However, in a cellular senescent model induced by cigarette smoke extract (CSE), more mitochondrial fragmentation was observed. In addition, mitochondrial fragmentation induction by knockdown of fusion proteins, OPA1 or MFN, increased mitochondrial ROS production and cellular senescence. For *in vivo* studies, inhibition of mitochondrial fission by deleting a mitochondrial fission protein DRP1 or maintenance of the fused mitochondrial network is necessary for longevity in yeast or *C. elegans* (Scheckhuber et al., 2007; Chaudhari and Kipreos, 2017). However, disruption of either mitochondrial fission or fusion significantly reduces medium lifespan in *C. elegans*, while in another study, promoting mitochondrial fission in midlife prolongs healthy lifespan of *D. melanogaster* (Rana et al., 2017; Byrne et al., 2019). Together, these conflicting data warrant more research to study the role of mitochondrial fusion or fission, or the imbalance of mitochondrial fusion/fission, in cellular senescence. The other mitochondrial quality control processes include mitochondrial biogenesis that propagates pre-existing pool of mitochondria, and mitophagy that eliminates malfunctional mitochondria. Mitochondrial biogenesis occurs constantly at basal levels and increases during cell renewal, development and stress conditions. Although in general mitochondrial biogenesis decreases during aging, expression of mitochondrial biogenesis regulators is upregulated in models of cellular senescence, which may reflect a compensatory response (Lee et al., 2002; Moiseeva et al., 2009). Mitophagy generally positively impacts lifespan and healthspan. Decreased mitophagy was observed during aging, while overexpression of Parkin and Pink1 extended lifespan in flies (Todd and Staveley, 2012; Rana et al., 2013; Bakula and Scheibye-Knudsen, 2020). Regarding mitophagy in senescence, mitophagy activities have been shown to be reduced in senescent cells *in vitro* and *in vivo* (Dalle Pezze et al., 2014; Garcia-Prat et al., 2016). This may be the consequence of reduced mitochondrial fission, autophagy and lysosomal activities, since mitochondrial fission is required for the separation of dysfunctional mitochondrial from the mitochondrial network, while lysosome is required for the terminal events of both autophagy and mitophagy.

Regarding the role of mitochondrial in RPE senescence, PGC-1α, a key protein for mitochondrial biogenesis and redox control, has been hypothesized to repress RPE senescence (Kaarniranta et al., 2018). We have recently reported that mitochondrial hyperfusion drives RPE senescence (Yu et al., 2020). We found that deletion of mitochondrial phosphatase phosphoglycerate mutase 5 (PGAM5) leads to accelerated RPE senescence *in vitro* and *in vivo*. Mechanistically, PGAM5 is required for mitochondrial fission through dephosphorylating DRP1. PGAM5 deletion leads to increased mitochondrial fusion and decreased mitochondrial turnover. As results, cellular ATP and ROS levels are elevated, mTOR and IRF/IFN-β signaling pathways are enhanced, leading to cellular senescence. Overexpression of DRP1-K38A mutant overexpression attenuated mitochondrial fission and elongated mitochondrial branches, which phenocopies *PGAM5* ^*−/−*^ senescent phenotypes, while overexpression of DRP1-S37A mutant dramatically inhibited mitochondria fusion and rescued mTOR activation and senescence in *PGAM5* ^*−/−*^ cells. These data support that mitochondrial dynamics can regulate signaling pathways linking to RPE senescence. In hydrogen peroxide (H_2_O_2_)-mediated premature RPE senescence model and natural passage-mediated replicative RPE senescence model, levels of ROS and mitochondrial membrane potential were increased, while coculture of RPE cells with embryonic stem cells reversed RPE senescence phenotypes *in vitro* (Wang et al., 2020). Cigarette smoke is one of the major risk factors for AMD. CSE can induce RPE senescence (Marazita et al., 2016). CSE increased superoxide while decreasing ATP production in RPE cells, suggesting an uncoupling of OXPHOS and mitochondrial dysfunction. Mice with intravitreal injection of CSE showed decreased TOM20 protein and ATP levels in the injected eyes (Cano et al., 2014). The exact function of mitochondria in CSE-induced RPE senescence is yet to be established.

## 7 Role of Mitochondria in Retinal Pigmented Epithelial Cell Death

Mitochondria also have significant involvement in different types of cell death. The role of mitochondria in apoptosis is well-established. Mitochondrial outer membrane permeabilization (MOMP) induced by B-cell lymphoma 2 (BCL-2) antagonist/killer (BAK) and BCL-2–associated X (BAX), causes the release of intermembrane space proteins including cytochrome c, second mitochondria-derived activator of caspase (SMAC) and Omi to the cytoplasm. Omi and SMAC promote caspase-9 activation by cleaving or binding to inhibitor of apoptosis proteins, while cytochrome c binds to apoptotic peptidase activating factor-1. These lead to apoptosome formation and caspase recruitment, caspase cascade activation and dismantling of the cellular contents (Vringer and Tait, 2019). Although apoptosis is generally believed to be immunotolerant, MOMP was recently shown to induce potent pro-inflammatory signaling. The role of mitochondria in necroptosis, a regulated form of necrosis, has been controversial (Marshall and Baines, 2014). Mitochondrial fragmentation has been observed in necroptosis (Wang et al., 2012). BAX and BAK function as the outer membrane component of the mitochondrial permeability pore (MPTP) and regulate MPTP opening through oligomerization during necrotic cell death (Karch et al., 2013; Karch et al., 2015). Numerous studies support the role of mitochondrial dysfunction, including mitochondrial ROS, activation of mitochondrial phosphatase PGAM5, and induction of mitochondrial permeability transition in necroptosis. However, recent studies suggest none of these are involved in necroptosis. Necroptosis was still functional in cells with mitochondria depleted by mitophagy (Tait et al., 2013). Further work is needed to confirm whether mitochondria are fully deleted in those cells and whether necroptosis is mitochondrial independent. During pyroptosis, rounded and fragmented mitochondria and mitochondrial outer membrane permeabilization were observed after the opening of gasdermin D (GSDMD)-dependent pores before the sudden rupture of the plasma membrane, and there is little evidence that mitochondria are involved in pyroptosis (de Vasconcelos et al., 2019). However, mitochondrial ROS was recently shown to mediate pyroptosis (Evavold et al., 2021). In that study, Ragulator-Rag complex controls mTORC1-dependent events to promote mitochondrial ROS, which operates downstream of GSDMD cleavage to promote GSDMD oligomerization and pore formation. The role of mitochondria in ferroptosis is still controversial (Gan, 2021). Diverse metabolic activities in mitochondria can facilitate ferroptosis, but mitochondria are also equipped with strong anti-ferroptosis defense systems. For example, ubiquinol (CoQH2) generated in mitochondria can defend against ferroptosis, but mitochondrial ROS and ATP can promote ferroptosis. Ferroptotic cells do not have typical necrotic morphological features, but mainly display mitochondrial shrinkage, increased mitochondrial membrane density and reduced mitochondrial cristae (Yagoda et al., 2007; Yang and Stockwell, 2008). However, cells lacking mitochondria are as sensitive to ferroptosis as parental cells with intact mitochondria, supporting the existence of mitochondrial independent ferroptosis (Dixon et al., 2012). Ferroptosis Suppressor Protein 1 (FSP1) was recently shown to exert strong anti-ferroptosis function on plasma membrane in the absence of glutathione peroxidase 4 (GPX4), supporting the existence of mitochondrial-independent ferroptosis (Bersuker et al., 2019; Doll et al., 2019).

Different kinds of RPE cell death have been reported depending on the types of stressors (Hanus et al., 2015; Tong and Wang, 2020). Mitochondrial changes have been associated with RPE cell death. N-retinylidene-N-retinyl-ethanolamine (A2E) is a by-product of the visual cycle formed by the reaction of two trans-retinal molecules with phosphatidyl-ethanolamine (Sparrow et al., 2003). It is a major fluorophore in lipofuscin and is accumulated in aging RPE (Sparrow and Boulton, 2005). A2E has been shown to induce RPE apoptosis by inhibiting cytochrome C oxidase, associated with declined mitochondrial activity and release of cytochrome c (Shaban et al., 2001). Blue light could potentially produce retinal toxicity leading to the development of degenerative eye diseases, such as AMD (Contin et al., 2013; Kuse et al., 2014). Blue light could lead to RPE cell necrosis or apoptosis, possibly depending on the intensity and duration of the light (Pang et al., 1999; Seko et al., 2001). In A2E-loaded RPE cells, blue light reduced cell viability, associated with decreased ATP and increased ROS levels (Marie et al., 2018; Alaimo et al., 2019). Blue light caused the imbalance of mitochondrial fusion/fission towards mitochondrial fragmentation in both non-loaded and A2E-loaded cells, which is correlated with decreased OPA1 and increased DRP1 expression levels. It was also showed that A2E treated cells led to an increase in both mitochondrial fusion and fission. Blue light induced mitochondrial fragmentation in A2E-loaded cells, consistent with their increased propensity to die. Exposure to UV radiation can induce oxidative stress and is associated with ocular pathologies (Marchitti et al., 2011). UV has been shown to induce RPE apoptosis (Yao et al., 2013). In UV-treated RPE cells, upregulation of cytochrome c protein and increased ROS production were observed, suggesting that mitochondrial membrane integrity was compromised (Balaiya et al., 2010). Bidirectional movement of short and elongated mitochondria was noted in control RPE cells. Immediately after UV irradiation, mitochondria became shorter and no longer moving, with very few branched mitochondria remaining within the cells (Bantseev and Youn, 2006; Youn et al., 2010). Loss of mitochondrial membrane potential and the number of mitochondria were also reported in UV-treated RPE cells (Youn et al., 2007; Hsieh et al., 2018). Menadione (or Vitamin K3) induces RPE apoptosis, associated with mitochondrial depolarization and cytochrome C release (Zhang et al., 2003). Exposure of RPE cells to a lethal dose of H_2_O_2_ (1 mM) has been shown to induce BAX translocation to the mitochondria and the release of apoptosis-inducing factor from the mitochondria (Ho et al., 2006).

Our lab has shown that H_2_O_2_ or tert-butyl hydroperoxide (tBHP) treatment induces RPE necrosis, which can be prevented by necrosis inhibitors necrostatins but not caspase inhibitor z-VAD (Hanus et al., 2013). Fragmentation and degeneration of mitochondrial network were observed in the treated cells. Sodium iodate (NaIO_3_) injection has been extensively used as a pre-clinical model of RPE dystrophy and GA (Wang J., 2013). Similar necrotic phenotypes were observed in NaIO_3_-treated RPE cells, which was associated with fragmented and clustered mitochondrial network in the perinuclear region (Hanus et al., 2016). Auranofin, an inhibitor of redox proteins TrxR1 and TrxR2, induced pyroptosis in RPE cells, which was repressed by NLRP3 and Caspase-1 inhibitors (Yumnamcha et al., 2019). In these cells, reduced ATP production and mitochondrial membrane potential, increased ROS, accumulation of damaged, fragmented and vesiculated mitochondria, and mitophagic flux to lysosomes, were observed. NaIO_3_ and high glucose have also been shown to induce RPE ferroptosis, which can be repressed by ferroptosis inhibitors (Liu et al., 2021; Tang et al., 2022). In an independent study, high glucose promoted RPE apoptosis and inhibited cell proliferation and mitophagy by inactivating ROS/Pink1/Parkin signal pathway (Zhang et al., 2019). Reduced mitochondrial membrane potential, increased ROS production were observed in NaIO_3_-treated cells, and reduced mitochondrial size and ridge, disrupted mitochondrial membrane were observed in high glucose-treated cells. Further study indicated that high glucose increased the expression of thioredoxin-interacting protein (TXNIP), which is associated with mitochondrial membrane depolarization, fragmentation and mitophagic flux to lysosomes. Elongated mitochondrial network was observed under low glucose, while fragmented mitochondria were observed in high glucose. TXNIP knockdown by shRNA prevented mitochondrial fragmentation and mitophagic flux under high glucose (Devi et al., 2019). Taken together, different stressors could induce different types of RPE cell death, mitochondrial dysfunction including reduced mitochondrial membrane potential, decreased ATP and increased ROS levels, and in many cases mitochondrial fragmentation and/or mitochondrial fission/fusion imbalance, were generally observed, supporting a role for mitochondria in RPE cell death.

## 8 Restoring Mitochondrial Function as Treatment Option for Retinal Pigmented Epithelial Aging and Age-Related Macular Degeneration

Given the critical role of mitochondrial function and dynamics in senescence and cell death, extensive efforts are being made to target mitochondria for RPE aging and age-related diseases, especially AMD. Here we summarize the current preclinical experiments and clinical studies on this topic (Table 3).

**TABLE 3:** Potential therapeutics for RPE Aging and AMD through restoring mitochondrial function.

Compound	Functions	References
Humanin	Reduce pro-apoptosis gene expression levels	Nashine et al. (2017)
	Prevent the loss of AMD mitochondria	
	Protect oxidative-stress induced RPE cell death and senescence	Sreekumar et al. (2016)
	Prevent oxidative stress-induced decrease in mitochondrial bioenergetics	
	Increase mitochondrial DNA copy number	
	Upregulate the expression of mitochondrial transcription factor A	
Resveratrol	Improve cell viability	(King et al., 2005; Sheu et al., 2010; Sheu et al., 2013; Nashine et al., 2020; Neal et al., 2020) (Borra et al., 2005)
	Decrease ROS level	
	Stimulate mitochondrial bioenergetics	
	Induce autophagy, pro-survival and specific anti-inflammatory response	Josifovska et al. (2020)
	Suppress choroidal neovascularization	Nagai et al. (2014)
	Activate SIRT1, a key regulator of cellular senescence, aging and longevity	Borra et al. (2005)
Chrysoeriol	Diminish mitochondrial dysfunction	Kim et al. (2021a)
	Prevent ROS accumulation	
	Enhance expression of anti-oxidative genes	
	Attenuate oxidative stress-induced mitochondrial membrane potential loss	
Necrostatins	Protect oxidative stress-induced RPE cell death *in vitro* and *in vivo*	(Hanus et al., 2015; Hanus et al., 2016)
	Recover mitochondrial dysfunction and reduce ROS production in response to necroptosis inducer TNFα or acetaminophen	(Ye et al., 2012; Takemoto et al., 2014)
PU-91	Upregulate PGC-1α	Nashine et al. (2019)
	Increase mtDNA copy number	
	Upregulate the genes involved in mitochondrial biogenesis pathway	
	Increase mitochondrial membrane potential	
	Decrease the level of mitochondrial superoxide	
	Upregulate SOD2 expression level	
TPP-Niacin	Ameliorate H_2_O_2_-induced Mitochondrial dysfunction and mitochondrial membrane potential reduction	Kim et al. (2021b)
	Enhance the expression of transcription factors (PGC-1α and NRF2) and antioxidant-associated genes (HO-1 and NQO-1)	
ZLN005	Upregulate of PGC-1α and its associated transcription factors, antioxidant enzymes, and mitochondrial genes	Satish et al. (2018)
	Increase basal and maximal respiration rates, and spare respiratory capacity	
AICAR, Metformin, Trehalose	Maintain RPE mitochondrial function by activating AMPK pathway and boost autophagy	(Zhao et al., 2020) (Ebeling et al., 2022)
Rapamycin	Inhibit mTOR and activate autophagy	(Huang et al., 2019; Go et al., 2020)
Nicotinamide mononucleotide (NMN)	Improve mitochondrial functions including basal respiration, maximal respiration, spare respiratory capacity and ATP production	Ebeling et al. (2020)
Elamipretide	Reduce RPE cell death and senescence. Under phase II clinical trail	Mettu et al. (2022)
α-Lipoic acid (LA)	Protect against an acute acrolein-induced RPE cell death	Jia et al. (2007)
	Prevent mitochondrial membrane potential decrease	
	Inhibit generation of intracellular oxidants	
	Prevent the intracellular SOD decrease	
	Protect mitochondrial complex I, II, and III activity	
	Increase intracellular total antioxidant power in RPE cells	
Melatonin	Protect human RPE cells against cytotoxic effects of H_2_O_2_	Rosen et al. (2012)
	Protect of mtDNA of ARPE-19 cells against H_2_O_2_-induced damage	Liang et al. (2004)
SkQ1	Prevent progression of retinopathy and suppressed atrophic changes in the RPE cells in the senescence-accelerated OXYS rats	(Muraleva et al., 2014; Muraleva et al., 2019; Telegina et al., 2020)

Abbreviations: RPE, retinal pigmented epithelial; AMD, Age-related macular degeneration; ROS, reactive oxygen species; POS, photoreceptor outer segments; BrM, Bruch’s membrane; IL, interleukin; TNF, tumor necrosis factor; IFN, interferon; TGF, transforming growth factor; TCA, tricarboxylic acid; ER, endoplasmic reticulum; ATP, adenosine triphosphate; mtDNA, mitochondrial DNA; NAD, nicotinamide adenine dinucleotide; GTPase, Guanosine triphosphatases; MFN, mitofusins; Opa1, Optic atrophy 1; Drp1, Dynamin-related protein 1; PGC, Peroxisome proliferator-activated receptor gamma coactivator; NRF, nuclear respiratory factors; TOM, translocase of the outer membrane; Pink1, PTEN-induced putative kinase 1; AMPK, Adenosine5′-monophosphate (AMP)-activated protein kinase; LC3, Microtubule-associated protein 1 light chain 3; TEM, transmission electron microscopy; MT/LT, MitoTracker/LysoTracker; PUFA, polyunsaturated fatty acids; UV, ultraviolet; EMT, Epithelial-mesenchymal transition; GA, geographic atrophy; SD-OCT, spectral domain optical coherence tomography; TUNEL, Terminal deoxynucleotidyl transferase dUTP, nick end labeling; iPSC, induced pluripotent stem cell; NLRP3, NLR, Family Pyrin Domain Containing 3; mtHsp, mitochondrial heat shock protein; CFH, Complement factor H; SIRT1, Sirtuin 1; mTOR, mammalian target of rapamycin; NRF2, Nuclear factor erythroid 2-related factor 2; OXPHOS, oxidative phosphorylation; Fis1, Mitochondrial fission 1 protein; CSE, cigarette smoke extract; PGAM5, Phosphoglycerate mutase 5; H2O2, hydrogen peroxide; ΔΨm, Mitochondrial membrane potential; MOMP, mitochondrial outer membrane permeabilization; BCL-2, B-cell lymphoma 2; BAK, BCL-2, antagonist/killer; BAX, BCL-2–associated X; SMAC, Second mitochondria-derived activator of caspase; MPTP, mitochondrial permeability pore; GSDMD, Gasdermin D; CoQH2, ubiquinol; FSP1, Ferroptosis Suppressor Protein 1; GPX4, Glutathione peroxidase 4; A2E, N-retinylidene-N-retinyl-ethanolamine; tBHP, tert-butyl hydroperoxide; NaIO3, sodium iodate; TXNIP, Thioredoxin-interacting protein; Nec-1, Necrostatin-1; RIPK1, Receptor Interacting Serine/Threonine Kinase 1; PPAR; Peroxisome proliferator-activated receptors; TPP, triphenylphosphonium; AICAR, 5-Aminoimidazole-4-carboxamide ribonucleotide; NMN, nicotinamide mononucleotide; LA, *α*-Lipoic acid; SkQ1, Plastoquinonyl-decyl-triphenylphosphonium.

1) **Humanin** is a mitochondrial-derived peptide with cytoprotective function in different disease models. Using cybrid containing AMD mitochondria, Nashine et al. found that humanin has a pivotal role in protecting cells with AMD mitochondria, reducing pro-apoptosis gene expression and increasing protection against amyloid-β-induced damage (Nashine et al., 2017). Mechanistically, humanin acts via both intracellular (BAX) and extracellular (gp130) pathways and prevents the loss of AMD mitochondria. In an independent study, humanin was shown to protect oxidative-stress induced RPE cell death and senescence (Sreekumar et al., 2016). Humanin treatment prevented oxidative stress-induced decrease in mitochondrial bioenergetics, increased mtDNA copy number and upregulated the expression of mitochondrial transcription factor A, a key biogenesis regulator protein. These studies suggest the potential for humanin therapy for prevention of retinal degeneration, including AMD.

2) **Resveratrol** is a phytoalexin synthesized by numerous plants including vines with strong antioxidative properties. Resveratrol improved cell viability and decreased ROS levels in AMD cybrid model and stimulated mitochondrial bioenergetics in RPE cells (King et al., 2005; Sheu et al., 2010; Sheu et al., 2013; Nashine et al., 2020; Neal et al., 2020). It also induced autophagy, pro-survival and specific anti-inflammatory response in RPE cells (Josifovska et al., 2020). In addition, resveratrol suppressed choroidal neovascularization in an animal model of wet AMD (Nagai et al., 2014). Other studies showed that resveratrol activates SIRT1, a key regulator of cellular senescence, aging and longevity (Borra et al., 2005). Based on these, resveratrol has great potential as therapeutic targets for RPE aging and AMD.

3) **Chrysoeriol** is a flavonoid compound that is commonly found in plants of the genus *Perilla frutescens.* This compound possesses several health-beneficial properties, including antioxidant (Mishra et al., 2003; Nickavar et al., 2016), anti-inflammatory (Choi et al., 2005) and anti-tumor activities (Min et al., 2020). Chrysoeriol protected H_2_O_2_-induced RPE cell death by diminishing mitochondrial dysfunction, preventing ROS accumulation and enhancing the expression of anti-oxidative genes including NRF2 (Kim et al., 2021a). It attenuated oxidative stress-induced MMP loss and upregulated mitochondrial related gene expression, including OXPHOS genes, mitochondrial process genes, and mtDNA replication and transcription genes. It also significantly increased TOM20, MFN2, and OPA1 expression and decreased H_2_O_2_-induced DRP1 expression and phosphorylation (at Ser 616 position). Further work is needed to directly test the role of chrysoeriol in mitochondrial biogenesis and quality control, as well as RPE aging and degeneration *in vivo.*

4) **Necrostatins** are inhibitors of necroptosis (Degterev et al., 2005). Necrostatin-1 (Nec-1) is the most common inhibitor of necrosis that targets receptor Interacting Serine/Threonine Kinase 1 (RIPK1); Nec-5 is the necrosis inhibitor that inhibits RIPK1 indirectly (Wang et al., 2007); while Nec-7 targets RIPK1-independent necrosis pathways (Zheng et al., 2008). Nec-1 has been shown to block necroptosis and ameliorate inflammatory response in multiple disease models (Cao and Mu, 2021). Our lab has shown that necrostatins protect oxidative stress-induced RPE cell death *in vitro* and *in vivo* (Hanus et al., 2015; Hanus et al., 2016). Other studies showed that Nec-1 can recover mitochondrial dysfunction and reduce ROS production in response to necroptosis inducer TNF-α or acetaminophen (Ye et al., 2012; Takemoto et al., 2014). Based on these, necrotstatins could have potential in regulating mitochondrial function and RPE degeneration, which awaits future work to confirm.

5) **PGC-1α enhancers**. PGC1-α is a critical regulator of mitochondrial biogenesis and redox control. PU-91, an FDA-approved mitochondrion-stabilizing drug, was shown to improve cell survival, mitochondrial health and anti-oxidative potential by upregulating PGC-1α in AMD cybrid model (Nashine et al., 2019). It increases mitochondrial DNA copy number, upregulates the genes involved in mitochondrial biogenesis pathway including PGC-1α, NRF-1, NRF-2, peroxisome proliferator-activated receptors (PPAR)-α, and PPAR-*γ*, increases mitochondrial membrane potential, decreases mitochondrial superoxides levels, upregulates SOD2 expression level and increases the production of mitochondrial derived peptides. Triphenylphosphonium (TPP) is a well-known mitochondrial targeting moiety. Vitamin B3 (niacin) is a powerful antioxidant with lipid lowering functions (Ganji et al., 2009; Boden et al., 2014). TPP-conjugated Niacin (TPP-Niacin) has been shown to improve cell viability, reduce ROS generation, and increase the antioxidant enzymes in H_2_O_2_-treated ARPE-19 cells (Kim et al., 2021b). It ameliorated H_2_O_2_-induced mitochondrial dysfunction and mitochondrial membrane potential reduction. It also markedly enhanced the expression of transcription factors (PGC-1α and NRF2) and antioxidant-associated genes (especially heme oxygenase-1 and NAD(P)H Quinone Dehydrogenase 1). ZLN005, a selective PGC-1α transcriptional regulator, protected RPE from cytotoxic oxidative damage (Satish et al., 2018). ZLN005-treated ARPE-19 cells showed robust upregulation of PGC-1α and its associated transcription factors, antioxidant enzymes, and mitochondrial genes, and enhanced mitochondrial function shown by increasing basal and maximal respiration rates, and spare respiratory capacity. In addition, ZLN005 protected ARPE-19 cells from cell death caused by H_2_O_2_, oxidized low-density lipoprotein, and NaIO_3_ without any cytotoxicity under basal conditions. ZLN005 protection effect is PGC-1α-dependent as it was lost in PGC-1α-silenced cells. Taken together, PGC-1α regulators, including PU-91, TPP-Niacin and ZLN005, could serve as novel therapeutic agents for RPE degeneration.

6) **Autophagy boosters (AMPK activators).** As autophagy (including mitophagy) has significant implications in aging and age-related diseases, autophagy regulators have been tested in RPE cells for protective response to oxidative stress. Metformin is the first-line anti-type 2 diabetes drug and has been known to stimulate autophagy in many tissues. In RPE cells, metformin conferred protection against H_2_O_2_-induced oxidative damage by activating AMPK pathway (Zhao et al., 2020). It also protected photoreceptors from light damage, delayed inherited retinal degeneration, and protected RPE from NaIO_3_-induced injury *in vivo* (Xu L et al., 2018). The protection was associated with decreased oxidative stress, decreased DNA damage, and increased mitochondrial energy production. A retrospective study indicates that metformin use is associated with decreased odds of developing AMD (Brown et al., 2019b). iPSC-RPE cells derived from AMD patients have been established to test the efficacy of drugs in AMD. Three AMPK activators AICAR (5-Aminoimidazole-4-carboxamide ribonucleotide), metformin, trehalose, have been tested for maintaining mitochondrial function in AMD iPSC-RPE cells (Ebeling et al., 2022). Rapamycin is a drug used to prevent organ transplant rejection. It inhibits mTOR and activates autophagy. Increased mTORC activity by RPE-specific deletion of mTOR suppressor tuberous sclerosis 1 led to RPE degeneration (Huang et al., 2019; Go et al., 2020). Inhibition of mTORC1 by rapamycin partially rescued RPE degeneration, supporting the potential therapeutic role for rapamycin in RPE degeneration.

7) **Mitochondrial metabolism regulators.** Reductive carboxylation is a major metabolic pathway in RPE cells (Du et al., 2016). It regulates cell viability, redox balance, mitochondrial function, and response to oxidative stress. Nicotinamide mononucleotide (NMN) is a key intermediate of NAD^+^, which is required to support reductive carboxylation and ATP production. NAD^+^ decreases with age. Oxidative stress depletes NAD^+^, and supplementation with NMN completely prevented RPE cell death induced by H_2_O_2_. Using RPE cells from AMD and control donors, only RPE cells from AMD donors show improvements in mitochondrial functions, including basal respiration, maximal respiration, spare respiratory capacity, and ATP production, after NMN treatment (Ebeling et al., 2020). Of note, NMN has been shown to mitigate age-related physiology decline in mice and is being pursued as anti-aging molecule in humans (Mills et al., 2016; Shade, 2020). These support a potential role of NMN in RPE aging and AMD.

8) **Elamipretide** is a mitochondria-targeted tetrapeptide that has been evaluated in mitochondrial diseases including primary mitochondrial myopathy and Barth syndrome (Sabbah, 2021). It acts by stabilizing cardiolipin and therefore increasing cellular ATP production and reducing mitochondrial ROS (Birk et al., 2014; Nickel et al., 2014; Szeto, 2014). In RPE-specific SOD2 knockout mice, daily topical Elamipretide treatment led to the prevention of RPE cell size increase, suggesting reduced RPE cell death and RPE senescence. Based on its mechanisms of action, phase 1 clinical trial has been conducted to evaluate its potential in dry AMD and noncentral GA after daily subcutaneous injection (Mettu et al., 2022). It appeared to be well tolerated without serious adverse effects. Phase 2A clinical trial is underway.

9) **α-Lipoic acid (LA)** is a mitochondria-targeted antioxidant and mitochondrial nutrient (Packer et al., 1997; Liu and Ames, 2005). Jia et al. reported that LA protected against acute acrolein (a toxicant present in cigarette smoke)-induced RPE cell death and mitochondrial membrane potential decrease. It also inhibited acrolein-induced generation of intracellular oxidants, prevented the intracellular SOD decrease, protected mitochondrial complex I, II, and III activity and increased intracellular total antioxidant power in RPE cells (Jia et al., 2007).

10) **Melatonin** protects mitochondria by scavenging ROS, inhibiting the MPTP, and activating uncoupling proteins. Thus, melatonin maintains the optimal mitochondrial membrane potential, preserves mitochondrial functions, and regulates mitochondrial biogenesis and dynamics. In most cases, melatonin reduces mitochondrial fission and elevates their fusion. It also has been found to promote mitophagy and improve homeostasis of mitochondria (Tan et al., 2016). In the retina, melatonin is released mainly by photoreceptor cells but can be also produced by other cell types in pathological conditions (Sakamoto et al., 2004). It has been reported that melatonin protects human RPE cells against the cytotoxic effects of H_2_O_2_ (Rosen et al., 2012). It was also shown to be effective in the protection of mtDNA of ARPE-19 cells against H_2_O_2_-induced damage (Liang et al., 2004). However, high exogenous concentrations of melatonin increase light-induced damage to photoreceptor (Tan et al., 2003). The antioxidant effect of melatonin may indicate its protective role in AMD.

11) **Plastoquinonyl-decyl-triphenylphosphonium (SkQ1)** is another mitochondrial targeted antioxidant. It has been reported that the treatment with SkQ1 (250 nmol/kg body weight) during the period of active disease progression (from 12 to 18 months of age) significantly prevented the progression of retinopathy and suppressed atrophic changes in the RPE cells in the senescence-accelerated OXYS rats (Muraleva et al., 2014; Muraleva et al., 2019; Telegina et al., 2020).

## 9 Conclusion Marks and Future Directions

RPE cells are critical for the metabolism and homeostasis of retina. Due to high metabolism, high exposure to light, oxidized POS and PUFAs, RPE cells are vulnerable to oxidative stress and other relevant stresses which make them more susceptible to aging and age-related disease. Mitochondria are the powerhouse of cells and can be a major source of cellular ROS that contribute to mtDNA damage, cell death, senescence, and age-related diseases. Mitochondria directly participate in cell death and senescence processes, and can undergo dynamic changes including fission/fusion, biogenesis and mitophagy for quality control in response to stresses. In this minireview, we described the RPE changes during aging and in AMD. The role of mitochondria in RPE aging and AMD was also discussed. Particularly, the involvement of mitochondria in RPE cellular senescence and death, two processes critical for RPE degeneration, and the current translational approaches to prevent RPE aging and degeneration, were summarized. Changes in the mitochondria of RPE, including mtDNA deletion and mutation, decreased ATP production, mitochondrial fission/fusion imbalance, decreased mitochondrial biogenesis and mitophagy and et al., were commonly observed during RPE aging and degeneration. More longitudinal studies, especially *in vivo* studies, are required to confirm some of the findings, since mitochondria could undergo dynamic changes during aging and in response to stresses, with adaptive response in the short term and pathological response in the long term. Based on the current research, although RPE senescence and cell death are involved in RPE aging and degeneration, the extent of their contribution and whether we can target RPE senescence and/or cell death for RPE aging and/or degeneration are still a matter of debate. Elimination of senescence using a senolytic approach increases mouse lifespan and has shown promise in human trials (Xu M et al., 2018; Justice et al., 2019). If RPE senescence is proved to be the major mechanism for RPE aging and degeneration, senolytic approach could be used to treat these conditions. Restoring mitochondrial function for preventing RPE aging and degeneration is an exciting idea, and many compounds listed above have shown promise in preclinical and clinical models. However, the understanding of mitochondria, especially mitochondrial dynamics and quality control, in RPE cell death and senescence, as well as RPE aging and degeneration, is still incomplete and awaits future studies. Stringent and new technologies, including genetic functional study, genetic lineage tracing, and single cell multi-omics studies, would be powered to answer these questions. New drug targets and drug candidates, more preclinical and clinical studies are also needed to test their efficacy and safety for RPE degeneration and AMD. Work on AMD-derived iPSC-RPE cells has shown considerable variability in drug response across patient cell lines (Ebeling et al., 2022). Therefore, a personalized medicine approach, including stratifying patients based on genotyping and more clinically relevant features, is needed in the future.
